# Dietary Fiber and Saturated Fat Intake Associations with Cardiovascular Disease Differ by Sex in the Malmö Diet and Cancer Cohort: A Prospective Study

**DOI:** 10.1371/journal.pone.0031637

**Published:** 2012-02-27

**Authors:** Peter Wallström, Emily Sonestedt, Joanna Hlebowicz, Ulrika Ericson, Isabel Drake, Margaretha Persson, Bo Gullberg, Bo Hedblad, Elisabet Wirfält

**Affiliations:** 1 Nutrition Epidemiology Research Group, Department of Clinical Sciences, Lund University, Malmö, Sweden; 2 Diabetes and Cardiovascular Disease, Genetic Epidemiology, Department of Clinical Sciences, Lund University, Malmö, Sweden; 3 Experimental Cardiovascular Research Unit, Department of Clinical Sciences, Lund University, Malmö, Sweden; 4 Internal Medicine Research Unit, Department of Clinical Sciences, Lund University, Malmö, Sweden; 5 Cardiovascular Epidemiology, Department of Clinical Sciences, Lund University, Malmö, Sweden; Indiana University School of Medicine, United States of America

## Abstract

**Background:**

The aim of the study was to examine associations between intake of macronutrients and dietary fiber and incident ischemic cardiovascular disease (iCVD) in men and women.

**Methods:**

We used data from 8,139 male and 12,535 female participants (aged 44–73 y) of the Swedish population-based Malmö Diet and Cancer cohort. The participants were without history of CVD and diabetes mellitus, and had reported stable dietary habits in the study questionnaire. Diet was assessed by a validated modified diet history method, combining a 7-d registration of cooked meals and cold beverages, a 168-item food questionnaire (covering other foods and meal patterns), and a 1-hour diet interview. Sociodemographic and lifestyle data were collected by questionnaire. iCVD cases, which included coronary events (myocardial infarctions or deaths from chronic ischemic heart disease) and ischemic strokes, were ascertained via national and local registries. Nutrient-disease associations were examined by multivariate Cox regressions.

**Results:**

During a mean follow-up of 13.5 years, we identified 1,089 male and 687 female iCVD cases. High fiber intakes were associated with lower incidence rates of iCVD in women and of ischemic stroke in men. In post-hoc analysis, we discovered statistically significant interactions between intake of fiber and saturated fat; these interactions also differed between men and women (p<0.001).

**Conclusions:**

In this well-defined population, a high fiber intake was associated with lower risk of iCVD, but there were no robust associations between other macronutrients and iCVD risk. Judging from this study, gender-specific nutrient analysis may be preferable in epidemiology.

## Introduction

Atherosclerosis is an important cause of cardiovascular disease (CVD). The classical lipid hypothesis states that high levels of cholesterol, particularly low density lipoprotein-cholesterol (LDL-C), in blood, leads to atherosclerosis. According to the same hypothesis, a high intake of saturated fat, particularly when combined with a low intake of polyunsaturated fat, will lead to increased LDL-C [1], [2]. However, several other cardiovascular disease processes have been identified (including chronic inflammation) [3]–[5], and many other dietary factors have been associated with CVD [6], implying that the lipid hypothesis is an insufficient explanation.

In addition, up until the previous decade, food and nutrient intakes were the analytic levels of choice in nutrition epidemiology, but more and more research on more aggregated levels is emerging. For example, although the support for a protective effect of monounsaturated fatty acids (MUFA) and a harmful effect of *trans*-fat were considered strong in the meta-analytic review on diet and CVD by Mente et al. [6], the strongest support of protection was found for “Mediterranean” and “prudent” dietary patterns and for foods such as vegetables and nuts, and avoidance of a “Western” dietary pattern and foods with a high glycemic index or load. Similarly, we have in the Malmö Diet and Cancer (MDC) cohort observed significant associations between food patterns, independently of estimated nutrient intakes, both with components of the metabolic syndrome (e.g. hyperglycemia, central obesity and hyperlipidemia, also risk factors of CVD) [7], and with inflammatory biomarkers [8].

In 2010, an FAO/WHO expert consultation issued recommendations on fat intake in humans [9], and recommended that total fat should constitute 20–35 percent of the total energy intake (energy percentage, E%) for maintenance of optimal micronutrient intakes and health. The evidence for total fat intake not being associated with increased coronary heart disease (CHD) risk was judged to be ‘probable’. Similarly, a high relative intake of saturated fatty acids (SFA) was not associated with increased CHD risk or CHD death in itself [10], although CHD risk was expected to diminish when SFA is replaced by polyunsaturated fatty acids (PUFA) [9]. Lack of support for a direct effect of saturated fat in epidemiological studies was also recently shown in meta-analyses by Mente et al. [6] and Siri-Tarino et al. [11]. Although it is difficult to examine the independent biological effect of nutrients found in the same foods (i.e., due to high co-linearity) [12], several researchers believe that studies based on data of high relative validity, partly or entirely derived from food records (such as the MDC cohort), rather than on the much more common food frequency questionnaires, may be more suitable for the study of closely associated nutrients, such as fatty acids and other components of macronutrients [13], [14].

We here present associations between intakes of macronutrients and fiber and incident ischemic CVD (i.e. coronary events or ischemic stroke, iCVD) in men and women with stable dietary habits from the population-based MDC cohort. We have previously shown that past food habit changers tend to report healthier food habits and lower energy intake compared with non-changers, but may still have excess risk of CVD due to their previous lifestyle. Further, they are more likely to be overweight and to be afflicted with other risk factors of CVD. Including them in the analysis may therefore distort observed diet-disease associations substantially [15], [16]. This project is partly an extension and update of an earlier paper [17] with four years of additional follow-up, inclusion of all non-alcohol macronutrients (as opposed to fat only), and a different analytical approach.

## Methods

### Study population

The MDC study is set in Malmö, Sweden's third largest city [18]. The background population consisted of all men born between 1923 and 1945 and all women born between 1923 and 1950 who were living in Malmö during the screening period 1991 to 1996 (n = 74,138). This population was identified through the Swedish national population registries. The final cohort consisted of 28,098 individuals (participation rate 40.8 percent). The subjects were recruited through advertisements in local media and through invitation by mail. The only exclusion criteria were inadequate Swedish language skills and mental incapacity [19], [20]. The Ethics Committee at Lund University approved the design of the MDC study (LU 51–90). Written informed consent was obtained from the participants. For the present study, we excluded all participants with prevalent cardiovascular disease (n = 855), self-reported diabetes (n = 798), and persons reporting previous dietary change (n = 5,761) (see “Other variables”, below). This left 20,674 persons available for analysis.

### Data collection

The study subjects visited the MDC study centre twice. At the first visit dietary interviewers provided information on the background and aim of the project, and detailed instructions about the dietary assessment and the other procedures of the study, including the lifestyle questionnaire. Height, weight and blood pressure were also measured by the study nurses. At the second visit, a dietary interview was performed (see below), and the lifestyle questionnaires were checked for incomplete answers.

### Dietary data

The MDC study used an interview-based, modified diet history method that combined (i) a 7-day menu-book for registration of lunch and dinner meals, cold beverages including alcohol, pharmaceutical drugs, natural remedies, and nutrient supplements, (ii) a 168-item questionnaire for assessment of meal pattern, consumption frequencies and portion sizes of regularly eaten foods, and (iii) a 1-hour interview. During the interview, the questionnaire and the menu book were checked, according to predefined rules, so that reported food consumption did not overlap and were in concordance with the overall meal pattern reported by the participant.

The mean daily intake of foods was calculated based on frequency and portion size estimates from the questionnaire and menu-book. The food intake was converted to energy and nutrient intakes using the MDC nutrient database, wherein the majority of the nutrient information comes from PC-KOST2-93 (National Food Administration, Uppsala, Sweden). The method is described in more detail elsewhere [21]. Data on the validity [22], [23] and reproducibility [24] of the method have been published.

In September 1994, the coding procedures of the dietary assessment were slightly altered in order to reduce interview time. When the alterations were evaluated, it was shown that the effects were very small [21]. We still chose to adjust for this alteration in the analyses, as described below.

### Dietary variables

The dietary variables used in this study were mean daily intakes of dietary total energy, total fat, saturated fatty acids (FA), monounsaturated FA, polyunsaturated FA, sums of ω3 FA (20:5+22:5+22:6+18:3), long-chain ω3 FA (20:5+22:6) and ω6 FA (20:4+18:2), total carbohydrates, monosaccharides, disaccharides, starch (computed as total carbohydrates minus the sum of mono- and disaccharides), dietary fiber, and protein. We calculated the gender-specific quintiles of energy percentages from these variables (non-alcohol energy), with the exception of dietary fiber, which was expressed as grams per 1000 kilocalories.

We also noted the season of the dietary interview: Winter (Dec–Feb), spring (Mar–May), summer (Jun–Aug) or autumn (Sep–Nov).

We further estimated misreporting of total energy intake, as described previously [25]. Briefly, total energy expenditure (TEE) was calculated using a multiple of calculated basal metabolic rate [26]. This multiple was estimated for each participant from questionnaire information on physical activity at work and during leisure time, household work, hours of sleep and other, more passive activities. Both duration and intensity of the activities were estimated, where applicable. The reported energy intake (EI) was compared with estimated TEE. Participants with reported EI:s outside a 95% confidence interval for TEE were considered low or high energy reporters [25], but were retained in the main analyses.

### Other variables

A structured multiple-choice questionnaire was used in the MDC study to collect information on sociodemographic factors, smoking status, alcohol habits, health status, use of pharmaceutical drugs, and several other factors. The agreement between the baseline questionnaire and the same questionnaire when repeated after three weeks was high for most variables (kappa values >0.75) [20].

The leisure time physical activity item was adapted from the Minnesota Leisure Time Physical Actitivity Questionnaire [27], [28]. The subjects were asked to fill in how many minutes per week they on the average were spending on the activity mentioned during each of the four seasons. These figures were all multiplied by an activity-specific factor. A physical activity score was obtained by computing the sum of all activity products. Four categories of physical activity status were identified by the subjects' quartile ranking (men and women separately).

We calculated body mass index (BMI) for each participant and categorized the results as suggested by the WHO [29]. Smoking status was divided into five categories: never-smoker, ex-smoker, 1–10, 11–20 or more than 20 cigarettes daily. Three percent of the current smokers had missing data on tobacco amount; they were coded as smoking 1–10 cigarettes daily. Education was split into four categories depending of length of education (< = 7 y, 8–9 y, 10–12 y, >12 y). Alcohol habits were classified as either none during last month, low (<20 g [men] or <15 g [women] alcohol daily), moderate (20–40 g [men] or 15–30 g [women]), or high (>40 g [men] or >30 g [women]). Other variables included systolic blood pressure (mm Hg), antihypertensive treatment (yes/no) and antihyperlipidemic treatment (yes/no). All persons answering yes to the question: “Have you ever been treated for diabetes?” were classified as diabetics, as were persons who reported using oral anti-diabetic drugs during the 7-day registration. Similarly, persons reported using antihyperlipidemic or antihypertensive drugs were classified as hyperlipidemics or hypertensives, respectively.

The main questionnaire item on change of dietary habits read “Have you ever substantially changed your dietary habits in the past?” with yes/no response categories. Particpants answering “yes” (n = 5,761) were excluded from the analysis, as noted above.

### Case ascertainment and follow-up

An ischemic cardiovascular event was defined as fatal or non-fatal myocardial infarction (MI) (ICD-9 codes: 410A–410X; ICD-10: I21), death from chronic ischemic heart disease (ICD-9 codes: 410–414; ICD-10: I20–I25), cerebral infarction (ischemic, ICD-9, code: 434; ICD-10: I63) or non-specific stroke (ICD-9, code: 436; ICD-10: I64). A coronary event (CE) was defined as either an MI or death from chronic ischemic heart disease. Subjects with intra-cerebral (ICD-9 code: 431; ICD-10: I61) or subarachnoid haemorrhages (ICD-9 code: 430; ICD-10: I60) were excluded from the analyses of stroke and total iCVD events. The reason for this was that risk factors of ischemic strokes are rather similar to those of CE, while those of haemorrhagic strokes are believed to be different. Ischemic strokes are associated with diabetes, high levels of total and low density lipoprotein cholesterol and triglycerides in serum, atrial fibrillation and previous CVD (MI, stroke or leg claudication). Haemorrhagic strokes, on the other hand, have shown positive associations with high alcohol intake, and protective associations between high total serum cholesterol and intracerebral haemorrhages have been reported [30], [31].

Each individual was followed until 31 December 2006, the date of the first CVD event (CE or ischemic or haemorrhagic stroke event), or death, whichever came first. One hundred and forty-one persons (0.7% of the study population) emigrated and were therefore lost to follow-up; they were censored on the date of emigration. Haemorrhagic stroke events were used for censoring because the risk of ischemic CVD increases substantially after a haemorrhagic stroke [32]. The records of patients with CVD were obtained by from the National Patient Register and the National Cause of Death Register [33], and the Stroke Register in Malmö [34]. The mean follow-up period was 13.6±2.1 years for women and 13.2±2.5 years for men. A total of 1,089 male and 687 female iCVD cases were identified during the follow-up period.

### Statistical analysis

We used Cox regressions to examine whether the intakes of the selected macronutrients were associated with risk of CE, ischemic stroke or iCVD. In the basic models, the nutrient intakes were adjusted for age, diet assessment method version, log of total energy intake (continuous) [35], and season of dietary interview. In the full models, the intakes were further adjusted for BMI category, smoking category, education, alcohol habits, systolic blood pressure, antihypertensive treatment (yes/no), antihyperlipidemic treatment (yes/no), quartiles of leisure time physical activity score and quintiles of energy-adjusted dietary fiber. These variables were selected from the literature. All analyses were performed for men and women separately due to possible gender differences in food selection and reporting, biology and psychosocial factors [36]. Due to missing values in the adjustment variables, another 215 persons were excluded from these analyses, leaving 20,459 persons.

It could be argued that increased blood pressure and hyperlipidemia are intermediates in the assumed pathway between diet and CVD, and that adjustment for these variables in multivariate analysis would therefore constitute over-adjustment. Because of this, we repeated all analyses of the full models without these variables. In order to further reduce misclassification, we also repeated these analyses after having excluded low and high energy reporters, as defined above.

## Results

### Background information and nutrient intakes

Men were older than women at baseline, which is an effect of the MDC inclusion criteria. Further, men had higher systolic and diastolic blood pressure and were much more likely to be overweight than women. Men were more likely to be ex-smokers, while women were more likely than men to be never-smokers (Table 1). Women had higher relative intakes of fiber, total carbohydrates and protein, while men had higher intakes of starch, total fat and all fat components (Table 2).

**Table 1:** Selected background characteristics of participants of the Malmö Diet and Cancer cohort with stable dietary habits.

Variable	Men, mean (SD)	Women, mean (SD)
Age (years)	58.9 (7.0)	57.2 (7.9)
Systolic blood pressure^a^ (mmHg)	143.5 (19.1)	138.9 (20.0)
Diastolic blood pressure^b^ (mm Hg)	88.0 (9.8)	83.9 (9.7)

Variable	Men, n (%)	Women, n (%)
**BMI**		
Underweight (<18.50)	44 (0.5)	185 (1.5)
Normal (18.50–24.99)	3,106 (38.2)	6,737 (53.8)
Overweight (25.00–29.99)	4,050 (49.8)	4,078 (32.6)
Obese (> = 30.00)	930 (11.4)	1,525 (12.2)
**Educational level**		
< = 7 yrs	3,712 (45.7)	4,905 (39.2)
8–9 yrs	1,615 (19.9)	3,846 (30.7)
10–12 yrs	991 (12.2)	856 (6.8)
>12 yrs	1,802 (22.2)	2,902 (23.2)
**Smoking category**		
Never-smokers	2,386 (29.3)	5,592 (44.6)
Ex-smokers	3,285 (40.4)	3,328 (26.6)
1–10 g/d	924 (11.4)	1,684 (13.4)
11–20 g/d	1,106 (13.6)	1,709 (13.6)
21- g/d	436 (5.4)	219 (1.7)
**Alcohol category (men/women)**		
None during last month	556 (6.8)	1,461 (11.7)
Low (0–20/0–15 g/d)	5,106 (62.7)	8,851 (70.6)
Moderate (20–40/15–30 g/d)	1,856 (22.8)	1,915 (15.3)
High (40–/30- g/d)	621 (7.6)	308 (2.5)
**Antihypertensive treatment**	1,221 (15.0)	1,728 (13.8)
**Antihyperlipidemic treatment**	124 (1.5)	90 (0.7)

a Numbers by variable: Blood pressure – 8,129 men and 12,514 women; BMI – 8,130 men and 12,525 women; educational level – 8,120 men and 12,509 women; smoking – 8,137 men and 12,532 women; other variables – 8,139 men and 12,535 women.

**Table 2:** Distribution of non-alcohol energy percentages from selected nutrients in participants of the Malmö Diet and Cancer cohort with stable dietary habits (medians).

	Sex									
	Men (n = 8,139)					Women (n = 12,535)				
	Quintiles					Quintiles				
	1	2	3	4	5	1	2	3	4	5
Carbohydrate	36.6	41.2	44.2	47.2	51.7	37.8	42.1	45.0	47.9	52.2
Monosaccharides	3.6	4.9	6.0	7.3	9.5	4.5	6.2	7.4	8.9	11.2
Disaccharides	7.4	10.0	11.9	14.1	17.5	8.8	11.2	12.9	14.9	18.2
Starch	20.0	23.1	25.3	27.7	31.3	19.1	21.9	23.8	25.9	29.1
Fiber^a^	5.8	7.1	8.2	9.3	11.4	6.5	8.1	9.3	10.6	12.9
Fat, total	33.0	37.4	40.3	43.5	48.1	32.0	36.2	39.1	42.1	46.5
Saturated fat	13.0	15.2	16.8	18.9	22.7	12.9	15.1	16.7	18.6	22.1
Monounsaturated fat	11.4	13.1	14.2	15.3	17.0	11.0	12.5	13.6	14.6	16.1
Polyunsaturated fat	4.5	5.5	6.2	7.1	8.5	4.3	5.1	5.8	6.6	8.0
n-3 fatty acids	0.70	0.86	0.99	1.14	1.40	0.67	0.82	0.94	1.08	1.34
Long-chain n-3 fatty acids	0.08	0.13	0.19	0.30	0.53	0.07	0.12	0.18	0.27	0.49
n-6 fatty acids	3.5	4.3	5.0	5.8	7.1	3.3	4.0	4.7	5.4	6.7
Protein	12.5	14.0	15.2	16.4	18.4	12.9	14.5	15.7	16.9	18.9

a Expressed as grams per 1000 kcal reported energy intake.

### Coronary events

Tables S1 and S2 show the results for coronary events in men and women, respectively. Although several nutrients were associated with risk of CE in the basic multivariate model, most notably monosaccharides and fiber in both men and women (both negatively), no nutrients retained significance in the full model, although the protective fiber association in women was of borderline significance (*p* for trend = 0.067). However, in the full model, a low intake of saturated fat in women was associated with higher risk of CE (*p* for trend = 0.037).

### Ischemic strokes

In the basic models describing risk of ischemic stroke (Tables S3 and S4), starch and fiber (both men and women), and total carbohydrates and monosaccharides (men only), was negatively associated with risk of ischemic stroke, while intake of total fat, saturated fat, and monounsaturated fat was associated with increased risk of stroke in men. Only the protective association with fiber in men (*p* for trend = 0.050) remained borderline significant after full adjustment.

### Ischemic CVD

Monosaccharides and starch were associated with lower risk of iCVD (CE+ischemic stroke) in the basic models, while fat, particularly monounsaturated fat, was associated with increased risk (Table S5, Tables 3, 4). After full adjustment, fiber intake was negatively and significantly associated with iCVD in women (24 percent lower risk in the highest intake quintile compared to the lowest, 95 percent confidence interval −3 to −41 percent, *p* for trend = 0.022), but no other significant associations were noted (Tables 3, 4).

**Table 3:** Risk of total ischemic cardiovascular disease in 12,535 women (687 cases)^a^ by intake of carbohydrates, fiber and protein (multivariate hazard ratios with 95% confidence intervals per quintile of energy-adjusted intake).

*Women (n = 12,535)*		*1 (n = 2,507)*	*2 (n = 2,507)*	*3 (n = 2,507)*	*4 (n = 2,507)*	*5 (n = 2,507)*	*P for trend*
**Carbohydrates**	c/py^b^	138/29,599	135/29,633	137/30,078	124/30,021	153/30,241	
	Basic^c^	1.00	0.91 (0.72–1.16)	0.88 (0.69–1.11)	0.75 (0.58–0.95)	0.90 (0.71–1.13)	0.14
	**Full** d	**1.00**	**1.02 (0.80–1.31)**	**1.08 (0.84–1.38)**	**0.89 (0.69–1.16)**	**1.18 (0.91–1.54)**	**0.48**
**Monosaccharides**	c/py	142/29,225	131/29,791	140/30,044	124/30,090	150/30,421	
	Basic	1.00	0.78 (0.61–0.99)	0.75 (0.59–0.95)	0.64 (0.50–0.81)	0.72 (0.58–0.91)	0.003
	**Full**	**1.00**	**0.96 (0.75–1.22)**	**1.00 (0.78–1.28)**	**0.89 (0.68–1.16)**	**1.10 (0.84–1.43)**	**0.67**
**Disaccharides**	c/py	129/29,908	124/29,982	126/30,195	136/29,940	172/29,546	
	Basic	1.00	0.85 (0.66–1.09)	0.76 (0.59–0.97)	0.81 (0.64–1.04)	1.06 (0.84–1.34)	0.55
	**Full**	**1.00**	**0.87 (0.68–1.12)**	**0.79 (0.62–1.02)**	**0.80 (0.62–1.03)**	**0.94 (0.74–1.19)**	**0.57**
**Starch**	c/py	181/29,551	146/29,818	116/29,931	128/29,782	116/30,491	
	Basic	1.00	0.79 (0.64–0.99)	0.65 (0.52–0.82)	0.74 (0.59–0.94)	0.68 (0.54–0.87)	0.001
	**Full**	**1.00**	**0.89 (0.71–1.11)**	**0.77 (0.61–0.99)**	**0.89 (0.69–1.13)**	**0.88 (0.68–1.15)**	**0.34**
**Fiber**	c/py	173/28,876	131/29,510	133/29,990	125/30,265	125/30,930	
	Basic	1.00	0.67 (0.53–0.84)	0.63 (0.50–0.79)	0.56 (0.44–0.71)	0.54 (0.42–0.68)	<0.001
	**Full**	**1.00**	**0.77 (0.61–0.97)**	**0.80 (0.64–1.02)**	**0.71 (0.56–0.91)**	**0.76 (0.59–0.97)**	**0.022**
**Protein**	c/py	168/29,838	127/30,261	124/30,002	128/29,766	140/29,705	
	Basic	1.00	0.76 (0.60–0.95)	0.79 (0.62–0.99)	0.83 (0.66–1.05)	0.92 (0.72–1.17)	0.66
	**Full**	**1.00**	**0.81 (0.64–1.03)**	**0.85 (0.67–1.08)**	**0.88 (0.69–1.12)**	**0.97 (0.76–1.24)**	**0.96**

a 12,402 women and 676 cases in the full model due to missing values.

b Cases/person years.

c Basic model: Adjusted for age, method version, total energy intake (continuous), and season.

d Full model: Adjusted for age, method version, total energy intake (continuous), season, BMI class, smoking category, education, alcohol category, systolic blood pressure, antihypertensive treatment, antihyperlipidemic treatment, leisure time physical activity (quartiles) and quintiles of energy-adjusted dietary fiber.

**Table 4:** Risk of total ischemic cardiovascular disease in 12,535 women (687 cases)^a^ by intake of fat (multivariate hazard ratios with 95% confidence intervals per quintile of energy-adjusted intake).

*Women (n = 12,535)*		*1 (n = 2,507)*	*2 (n = 2,507)*	*3 (n = 2,507)*	*4 (n = 2,507)*	*5 (n = 2,507)*	*P for trend*
**Fat, total**	c/py^b^	144/30,265	128/29,925	139/29,851	134/29,985	142/29,546	
	Basic^c^	1.00	0.91 (0.72–1.16)	1.06 (0.84–1.35)	1.07 (0.84–1.35)	1.15 (0.91–1.46)	0.12
	**Full** d	**1.00**	**0.86 (0.67–1.09)**	**0.95 (0.75–1.22)**	**0.89 (0.69–1.15)**	**0.86 (0.66–1.13)**	**0.44**
**Saturated fat**	c/py	145/30,428	135/29,989	133/29,976	131/29,697	143/29,483	
	Basic	1.00	0.96 (0.76–1.22)	0.98 (0.77–1.24)	1.00 (0.79–1.27)	1.11 (0.88–1.41)	0.38
	**Full**	**1.00**	**0.94 (0.74–1.19)**	**0.89 (0.69–1.14)**	**0.84 (0.64–1.08)**	**0.87 (0.66–1.14)**	**0.22**
**Monouns. fat**	c/py	138/30,141	130/30,084	126/29,822	135/29,797	158/29,727	
	Basic	1.00	0.97 (0.76–1.24)	0.95 (0.75–1.22)	1.09 (0.86–1.39)	1.28 (1.02–1.62)	0.019
	**Full**	**1.00**	**0.90 (0.71–1.15)**	**0.86 (0.67–1.11)**	**0.94 (0.73–1.22)**	**0.98 (0.76–1.27)**	**0.94**
**Polyuns. fat**	c/py	145/29,559	135/29,696	134/29,933	142/30,259	131/30,125	
	Basic	1.00	0.93 (0.73–1.17)	0.98 (0.78–1.24)	1.08 (0.85–1.36)	1.06 (0.84–1.34)	0.34
	**Full**	**1.00**	**0.90 (0.71–1.14)**	**0.94 (0.74–1.20)**	**1.04 (0.82–1.32)**	**0.94 (0.74–1.20)**	**0.91**
**n-3 fatty acids**	c/py	121/29,935	121/30,024	130/30,030	150/29,833	165/29,749	
	Basic	1.00	0.92 (0.71–1.18)	0.96 (0.75–1.23)	1.07 (0.84–1.36)	1.09 (0.86–1.38)	0.22
	**Full**	**1.00**	**0.91 (0.71–1.18)**	**0.92 (0.71–1.18)**	**1.02 (0.80–1.30)**	**1.03 (0.81–1.38)**	**0.50**
**Long-chain n-3**	c/py	109/29,809	117/29,815	133/30,046	154/29,937	174/29,965	
	Basic	1.00	0.86 (0.66–1.11)	0.87 (0.68–1.13)	0.98 (0.77–1.26)	0.99 (0.78–1.27)	0.51
	**Full**	**1.00**	**0.88 (0.68–1.15)**	**0.94 (0.72–1.21)**	**1.02 (0.80–1.32)**	**1.07 (0.83–1.37)**	**0.25**
**n-6 fatty acids**	c/py	145/29,437	154/29,642	130/29,982	131/30,206	127/30,306	
	Basic	1.00	1.14 (0.91–1.43)	0.98 (0.77–1.24)	1.08 (0.85–1.37)	1.11 (0.87–1.42)	0.59
	**Full**	**1.00**	**1.07 (0.85–1.35)**	**0.96 (0.76–1.22)**	**1.04 (0.81–1.32)**	**0.98 (0.76–1.25)**	**0.75**

a 12,402 women and 676 cases in the full model due to missing values.

b Cases/person years.

c Basic model: Adjusted for age, method version, total energy intake (continuous), and season.

d Full model: Adjusted for age, method version, total energy intake (continuous), season, BMI class, smoking category, education, alcohol category, systolic blood pressure, antihypertensive treatment, antihyperlipidemic treatment, leisure time physical activity (quartiles) and quintiles of energy-adjusted dietary fiber.

### Sensitivity analyses

After removing the variables on hypertension, systolic blood pressure and antihyperlipidemic treatment from the full model, we observed that fiber was weakly associated with lower risk of CE among women (*p* for trend = 0.051, data not shown). The association between saturated fat and CE in women was stronger (*p* for trend = 0.018), while the association between fiber and stroke in men was slightly attenuated (*p* for trend = 0.053). The other results were virtually unchanged.

After exclusion of low and high energy reporters (1,216 men and 2,379 women), fiber intake was negatively associated with CE in women (p = 0.011), and the protective effect of fiber on total iCVD in women was even stronger (p = 0.003, data not shown). Otherwise the results were essentially similar to the main findings, including those on saturated fat and CE risk.

### Post-hoc analyses

**Figure 1:**
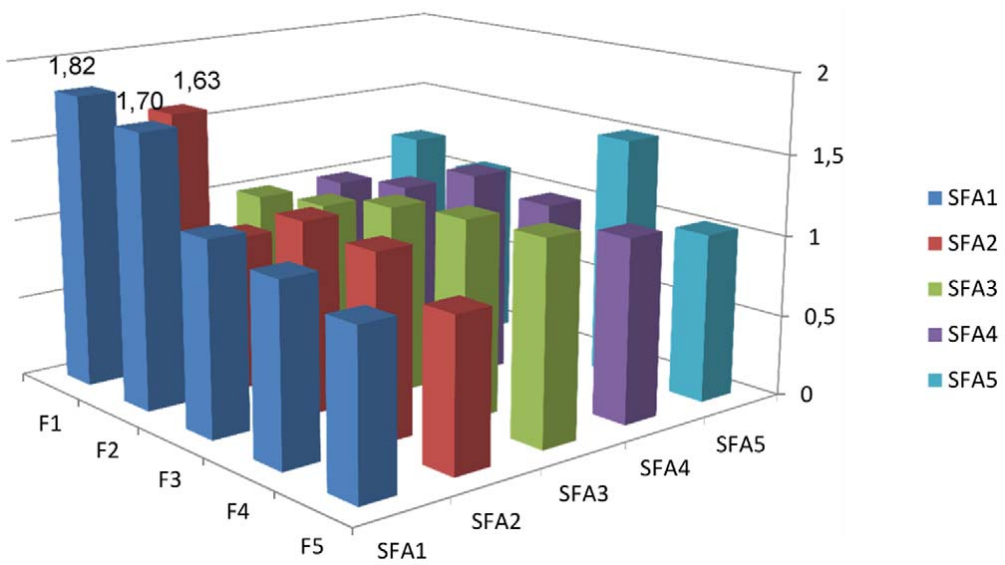
Joint effects of saturated fat and fiber intake on iCVD risk, men. Joint effects of quintiles of energy-adjusted saturated fat and fiber intake on risk of ischemic cardiovascular disease in men of the MDC cohort, expressed as hazard ratios. The numbers given in the figure are those significantly different (*p*<0.05) from the reference category (F5/SFA1). *p* value for the interaction between fiber and saturated fat = 0.041. Adjusted for age, method version, total energy intake (continuous), season, BMI class, smoking category, education, alcohol category, systolic blood pressure, antihypertensive treatment, antilipemic treatment and leisure time physical activity (quartiles). RR:s calculated with no individual nutrient variables in the model due to redundancy.

**Figure 2:**
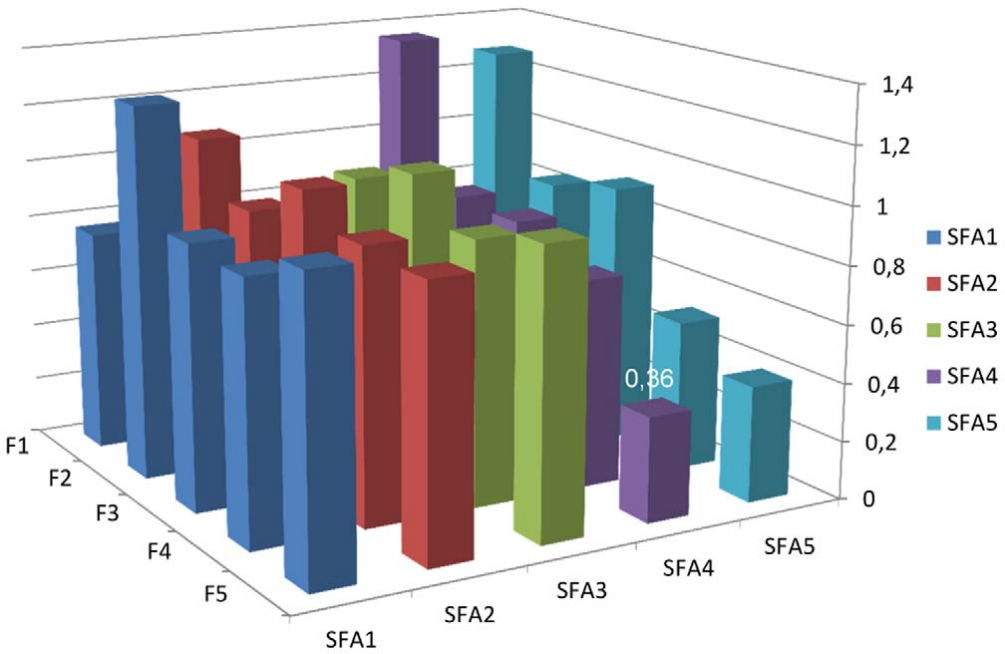
Joint effects of saturated fat and fiber intake on iCVD risk, women. Joint effects of quintiles of energy-adjusted saturated fat and fiber intake on risk of ischemic cardiovascular disease in women of the MDC cohort, expressed as hazard ratios. The numbers given in the figure are those significantly different (*p*<0.05) from the reference category (F5/SFA1). *p* value for the interaction between fiber and saturated fat = 0.003. Adjusted for age, method version, total energy intake (continuous), season, BMI class, smoking category, education, alcohol category, systolic blood pressure, antihypertensive treatment, antilipemic treatment and leisure time physical activity (quartiles). RR:s calculated with no individual nutrient variables in the model due to redundancy.

We also performed a post-hoc analysis in which we excluded fiber from the full model. The only difference was that the saturated fat-CE association in women was no longer significant (*p* for trend = 0.20). This prompted us to explore whether there were significant interactions between intakes of fiber and saturated fat. We used gender-specific nutrient quintiles, and the saturated fat, fiber and interaction terms were included simultaneously. In the full models, the interaction was statistically significant in women (*p*<0.001) vs. CE, but not in men (p = 0.065). Further, it was significant in women (*p* = 0.003) and men (*p* = 0.041) vs. iCVD, but not in the analysis of ischemic stroke. The joint effect of energy-adjusted saturated fat and dietary fiber on risk of iCVD is illustrated in Figures 1 and 2. Among men, most combinations of fiber and saturated fat entailed higher HRs than the reference category (highest fiber quintile and lowest SFA quintile), most pronounced in the lower intake ranges of both nutrients. Among women, however, the lowest HR's were seen in persons with high intakes of both nutrients.

An exploratory analysis of the full model with both men and women included revealed that the potential 3-way statistical interaction between gender, saturated fat and dietary fiber was strongly statistically significant for both CE and iCVD (*p* = 0.000032 and 0.00034, respectively). The interactions remained significant after exclusion of hypertension, systolic blood pressure and hyperlipidemia treatment from the full model (data not shown), and after exclusion of low and high energy reporters, although the interaction in women in the iCVD analysis was attenuated (*p* = 0.059, data not shown).

## Discussion

This study showed that although intake of several macronutrients were associated with increased or decreased risk of ischemic CE, stroke or iCVD, the lower risk of iCVD associated with a high fiber intake among women was clearly the most consistent and robust in multivariate analyses. There was also a borderline protective association between fiber and ischemic stroke among men. Among women, there was a protective association between fiber and CE after exclusion of low and high energy reporters. There was also a protective association between saturated fat intake and CE among women; this association, however, was dependent on fiber being present in the statistical model. Indeed, we discovered statistical interactions between intake of fiber and saturated fat, which also were different between men and women.

This study is largely consistent with several studies suggesting a protective effect of dietary fiber on CVD risk and CVD death [6], [37], [38]. Other researchers have noted that the effects of fiber may vary by source. For example, Pereira et al. noted that cereal and fruit fiber was associated with lower risk of coronary death [37]. At this time, it is not possible to analyze the food sources of nutrients on an individual basis in the MDC cohort. However, on average, women of the MDC cohort obtained 23.5 percent of their fiber intake from fruit and berries, 23.7 percent from vegetables (including potatoes and other tubers, carrots and legumes), and another 9.5 percent from crisp bread, while the corresponding figures in men were 15.7, 22.9, and 8.0 percent, respectively. The proportions of fiber from other cereals were very similar (5.4 and 5.2 percent, respectively). The difference in fiber sources between genders was mainly made up by a higher consumption of soft bread in men (20.4 vs 29.8 percent; all unpublished data). This may help explain the difference in the fiber-iCVD associations between men and women, particularly since women also had a higher relative fiber intake than men (Table 2).

This study provides little support for independent effects of specific macronutrients in the causation of ischemic CVD. Saturated fat, long suspected as a causal risk factor of CVD, was generally not associated with disease, although the women with the lowest intake had higher risk of CE than other women – after adjustment for fiber (Table S2). This illustrates one of the major problems with studies of nutrient intake: the nutrient variables are also, perhaps even primarily, markers of the foods they derive from [39]. Foods contain many nutrients and other bioactive substances that interact in complex ways and may therefore differ in their health effects in ways not captured by differences in the content of single nutrients. Also, the foods delivering the same amount of nutrient will vary between and within populations. For example: dairy products are in Sweden important sources of both saturated fatty acids (SFA) and MUFA. Further, dairy products are more important sources of SFA among women of the MDC cohort than among men, while meat is a correspondingly more important source among men (unpublished data). Like many other studies, our study suffers from relatively high correlations between some nutrients. The energy-adjusted correlation between the SFA and MUFA quintiles was around 0.57, with a correlation of the underlying continuous data of approximately 0.63. This is of course partly due to the fact that these nutrients in Sweden mostly originate from the same sources, i.e. dairy products and meat products. It is difficult to fully correct for associations such as these. There are thus good reasons to argue that epidemiologists should examine food intakes, dietary patterns or other more or less aggregated exposures in addition to nutrients [39]. Further, one should note that only 1.2 percent of the present study population actually followed national Swedish recommendations (less than 10 energy percent) on saturated fat intake. Strictly speaking, the SFA-CVD hypothesis is thus not fully testable in this population. On the other hand, fiber intake in Sweden is generally low, compared to other European countries [40]. It is therefore noteworthy that the apparent effects of higher fiber intake are rather strong in the present study.

The fiber-SFA interactions are not easily explained. We do not know of any experimental evidence giving any clues to potential biological mechanisms that would be involved to produce a protective effect of SFA, although other Swedish researchers recently did note a protective association between a high consumption of dairy products and the risk of CVD [41], as did our group [42]. If this effect were to be causal, it would thus probably be due to some component of milk other than SFA. Further, there was no protective effect of SFA on iCVD risk neither in men, nor in women, when inadequate energy reporters were excluded and fiber was not included in the multivariate model (*p* for trend = 0.80 in both genders). It is possible that our results are caused either by erroneous dietary reporting we were unable to control for, or by residual confounding, perhaps as a result of the same nutrient being consumed in the form of different foods. In addition, the fiber-SFA interactions differed by gender. Although men and women may be biologically different in ways that are relevant in the present context, it is probably more likely that the diverging results are due either to gender-associated differences in dietary habits, or to the reporting of them [36]. As always, the possibility of chance findings can never entirely be ruled out. Either way, the practice of combining nutrient data from men and women in epidemiology may be questioned. Confirmation of this finding from other researchers would be welcome.

One weakness of the study is the lack of information on *trans*-fatty acids (TFA) in the MDC database; these fatty acids are thus mainly included among the monounsaturated fatty acids. However, the levels of TFA in Swedish foods have been lowered considerably since the mid-1990s. The TFA intake in Sweden is now similar (i.e., very low) to that of Denmark, where TFA levels in foods have been strictly regulated [43].

We observed no differences in health outcomes with any of the carbohydrate subclasses. Although several carbohydrate variables were strongly significant in the less adjusted models, it is possible that the traditional carbohydrate division used in the MDC cohort is not biologically relevant [44]. Instead, many researchers in observational epidemiology currently attempt to estimate the effects of carbohydrate sources or other higher-level variables. Examples include whole grains, which appear to be protective beyond the associated fiber content [44]; dietary glycemic index and/or glycemic load [45], [46]; and food pattern and/or dietary index methods [44] (Hlebowicz & Drake et al, personal communication).

Recently, researchers have increasingly used substitution methods to analyze macronutrient data. Specifically, several studies have shown that substitution of SFA by carbohydrates and/or MUFA [46], [47] or PUFA [48] may alter relative risks of CVD. In our data, MUFA was strongly correlated with carbohydrates, SFA and to some extent with PUFA. In addition, the Spearman correlation coefficient between the saturated fat and fiber quintiles was −0.49 in both men and women. Further, the fiber-SFA interaction made SFA appear protective against CE in women. This may violate the assumption of most recent substitution analyses that SFA, if anything, are associated with increased risk. This might have made any differences detected by a substitution model more difficult to interpret. We thus decided to perform a more traditional analysis.

The strengths of this study include the high-quality dietary data [13], [14], [49], the size of the population-based cohort, the 99.3% complete follow-up, the high-quality case ascertainment and the inclusion of persons with stable dietary habits only, the latter being an advantage few comparable studies have. The importance of good quality confounder data may be appreciated by considering the differences between the results of the basic and the more fully adjusted models. It may be noted that BMI, smoking, education, alcohol habits, blood pressure and hyperlipidemia were all significantly associated with iCVD risk (data not shown). Weaknesses (in addition to those already mentioned) include the facts that we only had one dietary measurement and no available biomarkers of intake.

In summary, this study shows that a high fiber intake may lower the risk of CVD in general, although the evidence is stronger in women than in men. This study of a well-defined population, where SFA intake was high overall, provides little support for independent effects of specific macronutrients in relation to risk of ischemic CVD. However, we observed a 3-way interaction between gender, dietary fiber and saturated fat, supporting the idea that gender-specific nutrient analysis is preferable in epidemiolog.

## Supporting Information

Table S1 **Risk of coronary event in 8,139 men (688 cases) by macronutrient intake (multivariate hazard ratios with 95% confidence intervals per quintile of energy-adjusted intake).**
(DOC)

Table S2 **Risk of coronary event in 12,535 women (333 cases) by macronutrient intake (multivariate hazard ratios with 95% confidence intervals per quintile of energy-adjusted intake).**
(DOC)

Table S3 **Risk of ischemic stroke in 8,135 men (401 cases) by macronutrient intake (multivariate hazard ratios with 95% confidence intervals per quintile of energy-adjusted intake).**
(DOC)

Table S4 **Risk of ischemic stroke in 12,535 women (354 cases) by macronutrient intake (multivariate hazard ratios with 95% confidence intervals per quintile of energy-adjusted intake).**
(DOC)

Table S5 **Risk of total ischemic cardiovascular disease in 8,139 men (1089 cases) by macronutrient intake (multivariate hazard ratios with 95% confidence intervals per quintile of energy-adjusted intake).**
(DOC)
