# Supplementary material for: Dietary Fiber and Saturated Fat Intake Associations with Cardiovascular Disease Differ by Sex in the Malmö Diet and Cancer Cohort: A Prospective Study
Source: PLoS One. 2012 Feb 27;7(2):e31637. doi: 10.1371/journal.pone.0031637 (PMC3288044; doi:10.1371/journal.pone.0031637)
Supplement: Table S2 — Risk of coronary event in 12,535 women (333 cases) by macronutrient intake (multivariate hazard ratios with 95% confidence intervals per quintile of energy-adjusted intake). (DOC) [file pone.0031637.s002.doc]

Table S2.

|  |  | ***1 (n=2,507)*** | ***2 (n=2,507)*** | ***3 (n=2,507)*** | ***4 (n=2,507)*** | ***5 (n=2,507)*** | ***P for trend*** |
| --- | --- | --- | --- | --- | --- | --- | --- |
| **Carbohydrates** | c / py[[1]](#footnote-2) | 71 / 29,599 | 61 / 29,633 | 65 / 30,078 | 61 / 30,021 | 75 / 30,241 |  |
|  | Basic**[[2]](#footnote-3)** | 1.00 | 0.80 (0.57-1.13) | 0.82 (0.58-1.14) | 0.73 (0.52-1.02) | 0.88 (0.63-1.21) | 0.36 |
|  | **Full[[3]](#footnote-4)** | **1.00** | **0.91 (0.64-1.28)** | **1.01 (0.71-1.44)** | **0.88 (0.61-1.26)** | **1.17 (0.81-1.68)** | **0.52** |
| **Monosaccharides** | c / py | 74 / 29,225 | 66 / 29,791 | 73 / 30,044 | 50 / 30,090 | 70 / 30,421 |  |
|  | Basic | 1.00 | 0.76 (0.55-1.06) | 0.77 (0.55-1.06) | 0.50 (0.35-0.72) | 0.67 (0.48-0.93) | 0.003 |
|  | **Full** | **1.00** | **0.95 (0.68-1.34)** | **1.02 (0.72-1.43)** | **0.70 (0.48-1.04)** | **1.01 (0.69-1.47)** | **0.58** |
| **Disaccharides** | c / py | 57 / 29,908 | 57 / 29,982 | 64 / 30,195 | 70 / 29,940 | 85 / 29,546 |  |
|  | Basic | 1.00 | 0.89 (0.61-1.28) | 0.88 (0.61-1.26) | 0.96 (0.67-1.37) | 1.21 (0.86-1.70) | 0.18 |
|  | **Full** | **1.00** | **0.89 (0.61-1.29)** | **0.89 (0.62-1.28)** | **0.91 (0.64-1.30)** | **1.00 (0.70-1.41)** | **0.88** |
| **Starch** | c / py | 86 / 29,551 | 72 / 29,818 | 57 / 29,931 | 55 / 29,782 | 63 / 30,491 |  |
|  | Basic | 1.00 | 0.83 (0.61-1.14) | 0.68 (0.48-0.95) | 0.68 (0.48-0.96) | 0.79 (0.57-1.10) | 0.066 |
|  | **Full** | **1.00** | **0.95 (0.69-1.31)** | **0.86 (0.60-1.22)** | **0.88 (0.62-1.27)** | **1.10 (0.76-1.61)** | **0.88** |
| **Fiber** | c / py | 87 / 28,876 | 71 / 29,510 | 59 / 29,990 | 55 / 30,265 | 61 / 30,930 |  |
|  | Basic | 1.00 | 0.73 (0.53-1.00) | 0.56 (0.40-0.79) | 0.50 (0.36-0.71) | 0.54 (0.38-0.75) | <0.001 |
|  | **Full** | **1.00** | **0.84 (0.62-1.17)** | **0.73 (0.52-1.03)** | **0.65 (0.46-0.93)** | **0.78 (0.55-1.11)** | **0.067** |
| **Fat, total** | c / py | 71 / 30,265 | 62 / 29,925 | 65 / 29,851 | 60 / 29,985 | 75 / 29,546 |  |
|  | Basic | 1.00 | 0.89 (0.63-1.25) | 0.99 (0.70-1.39) | 0.94 (0.66-1.33) | 1.19 (0.85-1.66) | 0.29 |
|  | **Full** | **1.00** | **0.81 (0.57-1.14)** | **0.88 (0.62-1.24)** | **0.77 (0.53-1.12)** | **0.87 (0.60-1.28)** | **0.52** |
| **Saturated fat** | c / py | 82 / 30,428 | 58 / 29,989 | 63 / 29,976 | 59 / 29,697 | 71 / 29,483 |  |
|  | Basic | 1.00 | 0.72 (0.52-1.02) | 0.80 (0.57-1.12) | 0.77 (0.55-1.09) | 0.93 (0.67-1.30) | 0.77 |
|  | **Full** | **1.00** | **0.68 (0.48-0.95)** | **0.68 (0.48-0.96)** | **0.60 (0.41-0.86)** | **0.67 (0.46-0.97)** | **0.037** |
| **Monouns. fat** | c / py | 67 / 30,141 | 64 / 30,084 | 61 / 29,822 | 59 / 29,797 | 82 / 29,727 |  |
|  | Basic | 1.00 | 0.98 (0.69-1.38) | 0.93 (0.66-1.32) | 0.96 (0.68-1.37) | 1.34 (0.96-1.86) | 0.11 |
|  | **Full** | **1.00** | **0.90 (0.64-1.28)** | **0.83 (0.58-1.19)** | **0.82 (0.57-1.20)** | **1.02 (0.71-1.46)** | **0.99** |
| **Polyuns. fat** | c / py | 62 / 29,559 | 68 / 29,696 | 70 / 29,933 | 63 / 30,259 | 70 / 30,125 |  |
|  | Basic | 1.00 | 1.09 (0.77-1.54) | 1.18 (0.84-1.67) | 1.10 (0.78-1.57) | 1.30 (0.92-1.84) | 0.17 |
|  | **Full** | **1.00** | **1.04 (0.74-1.48)** | **1.13 (0.80-1.60)** | **1.09 (0.76-1.55)** | **1.19 (0.84-1.69)** | **0.32** |
| **n-3 fatty acids** | c / py | 51 / 29,935 | 58 / 30,024 | 64 / 30,030 | 80 / 29,833 | 80 / 29,749 |  |
|  | Basic | 1.00 | 1.05 (0.72-1.53) | 1.12 (0.78-1.63) | 1.36 (0.96-1.94) | 1.28 (0.90-1.82) | 0.062 |
|  | **Full** | **1.00** | **1.02 (0.70-1.48)** | **1.05 (0.72-1.52)** | **1.27 (0.89-1.81)** | **1.20 (0.84-1.71)** | **0.14** |
| **Long-chain n-3** | c / py | 55 / 29,809 | 57 / 29,815 | 61 / 30,046 | 76 / 29,937 | 84 / 29,965 |  |
|  | Basic | 1.00 | 0.85 (0.58-1.23) | 0.82 (0.57-1.19) | 1.00 (0.70-1.42) | 1.00 (0.71-1.42) | 0.55 |
|  | **Full** | **1.00** | **0.90 (0.62-1.31)** | **0.89 (0.62-1.29)** | **1.07 (0.75-1.52)** | **1.10 (0.77-1.57)** | **0.31** |
| **n-6 fatty acids** | c / py | 65 / 29,437 | 75 / 29,642 | 66 / 29,982 | 59 / 30,206 | 68 / 30,306 |  |
|  | Basic | 1.00 | 1.22 (0.88-1.71) | 1.10 (0.78-1.54) | 1.06 (0.74-1.52) | 1.30 (0.92-1.83) | 0.34 |
|  | **Full** | **1.00** | **1.18 (0.84-1.65)** | **1.09 (0.77-1.54)** | **1.06 (0.74-1.51)** | **1.19 (0.84-1.68)** | **0.55** |
| **Protein** | c / py | 78 / 29,838 | 63 / 30,261 | 65 / 30,002 | 59 / 29,766 | 68 / 29,705 |  |
|  | Basic | 1.00 | 0.82 (0.58-1.14) | 0.90 (0.64-1.25) | 0.84 (0.59-1.19) | 0.99 (0.70-1.40) | 0.97 |
|  | **Full** | **1.00** | **0.92 (0.65-1.29)** | **1.00 (0.71-1.40)** | **0.92 (0.64-1.31)** | **1.10 (0.77-1.56)** | **0.69** |

1. Cases / person years. [↑](#footnote-ref-2)
2. Basic model: Adjusted for age, method version, total energy intake (continuous), and season. [↑](#footnote-ref-3)
3. Full model: Adjusted for age, method version, total energy intake (continuous), season, BMI class, smoking category, education, alcohol category, systolic blood pressure, antihypertensive treatment, antihyperlipidemic treatment, leisure time physical activity (quartiles) and quintiles of energy-adjusted dietary fiber. There are only 12,402 women and 330 cases in the full model due to missing values. [↑](#footnote-ref-4)
