# Supplementary material for: Dietary Fiber and Saturated Fat Intake Associations with Cardiovascular Disease Differ by Sex in the Malmö Diet and Cancer Cohort: A Prospective Study
Source: PLoS One. 2012 Feb 27;7(2):e31637. doi: 10.1371/journal.pone.0031637 (PMC3288044; doi:10.1371/journal.pone.0031637)
Supplement: Table S5 — Risk of total ischemic cardiovascular disease in 8,139 men (1089 cases) by macronutrient intake (multivariate hazard ratios with 95% confidence intervals per quintile of energy-adjusted intake). (DOC) [file pone.0031637.s005.doc]

Table S5.

|  |  | ***1 (n=1,627)*** | ***2 (n=1,628)*** | ***3 (n=1,628)*** | ***4 (n=1,628)*** | ***5 (n=1,628)*** | ***P for trend*** |
| --- | --- | --- | --- | --- | --- | --- | --- |
| **Carbohydrates** | c / py[[1]](#footnote-2) | 214 / 18,706 | 225 / 18,688 | 222 / 18,945 | 212 / 18,940 | 216 / 18,922 |  |
|  | Basic**[[2]](#footnote-3)** | 1.00 | 0.98 (0.82-1.19) | 0.96 (0.80-1.16) | 0.86 (0.71-1.04) | 0.90 (0.74-1.09) | 0.12 |
|  | **Full[[3]](#footnote-4)** | **1.00** | **1.09 (0.90-1.32)** | **1.12 (0.92-1.36)** | **1.04 (0.85-1.28)** | **1.09 (0.88-1.35)** | **0.63** |
| **Monosaccharides** | c / py | 229 / 18,408 | 233 / 18,830 | 211 / 19,053 | 217 / 18,963 | 199 / 18,947 |  |
|  | Basic | 1.00 | 0.91 (0.76-1.10) | 0.74 (0.62-0.90) | 0.74 (0.62-0.90) | 0.69 (0.57-0.84) | <0.001 |
|  | **Full** | **1.00** | **1.03 (0.85-1.24)** | **0.91 (0.74-1.11)** | **0.95 (0.77-1.16)** | **0.91 (0.73-1.14)** | **0.31** |
| **Disaccharides** | c / py | 195 / 19,205 | 208 / 19,043 | 211 / 19,037 | 236 / 18,646 | 239 / 18,270 |  |
|  | Basic | 1.00 | 1.02 (0.84-1.24) | 1.00 (0.82-1.22) | 1.10 (0.91-1.34) | 1.13 (0.93-1.37) | 0.13 |
|  | **Full** | **1.00** | **1.07 (0.88-1.31)** | **1.04 (0.85-1.27)** | **1.11 (0.91-1.35)** | **1.04 (0.85-1.27)** | **0.66** |
| **Starch** | c / py | 245 / 18,312 | 216 / 18,657 | 225 / 18,900 | 193 / 19,086 | 210 / 19,246 |  |
|  | Basic | 1.00 | 0.86 (0.72-1.03) | 0.90 (0.75-1.08) | 0.77 (0.64-0.93) | 0.88 (0.73-1.06) | 0.083 |
|  | **Full** | **1.00** | **0.94 (0.78-1.14)** | **0.99 (0.82-1.21)** | **0.94 (0.76-1.16)** | **1.10 (0.88-1.37)** | **0.50** |
| **Fiber** | c / py | 246 / 18,095 | 219 / 18,714 | 213 / 18,916 | 220 / 18,972 | 191 / 19,504 |  |
|  | Basic | 1.00 | 0.84 (0.70-1.01) | 0.78 (0.65-0.94) | 0.79 (0.66-0.95) | 0.65 (0.54-0.79) | <0.001 |
|  | **Full** | **1.00** | **0.93 (0.78-1.12)** | **0.93 (0.77-1.12)** | **1.00 (0.82-1.20)** | **0.85 (0.70-1.04)** | **0.30** |
| **Fat, total** | c / py | 211 / 19,060 | 218 / 18,736 | 203 / 19,002 | 226 / 18,813 | 231 / 18,589 |  |
|  | Basic | 1.00 | 1.04 (0.86-1.25) | 0.97 (0.80-1.18) | 1.10 (0.91-1.34) | 1.18 (0.98-1.43) | 0.071 |
|  | **Full** | **1.00** | **0.99 (0.82-1.20)** | **0.92 (0.75-1.13)** | **1.01 (0.82-1.24)** | **0.95 (0.77-1.18)** | **0.77** |
| **Saturated fat** | c / py | 217 / 19,010 | 221 / 19,027 | 209 / 19,065 | 212 / 18,743 | 230 / 18,355 |  |
|  | Basic | 1.00 | 0.99 (0.82-1.20) | 0.97 (0.80-1.17) | 1.01 0.83-1.22) | 1.08 (0.89-1.30) | 0.4 |
|  | **Full** | **1.00** | **0.94 (0.78-1.14)** | **0.94 (0.77-1.15)** | **0.91 (0.74-1.12)** | **0.89 (0.72-1.11)** | **0.32** |
| **Monouns. fat** | c / py | 201 / 18,996 | 216 / 18,759 | 218 / 18,947 | 234 / 18,818 | 220 / 18,681 |  |
|  | Basic | 1.00 | 1.04 (0.86-1.26) | 1.07 (0.88-1.30) | 1.15 (0.96-1.40) | 1.16 (0.96-1.40) | 0.064 |
|  | **Full** | **1.00** | **0.97 (0.80-1.18)** | **1.00 (0.82-1.23)** | **1.02 (0.84-1.25)** | **0.92 (0.74-1.14)** | **0.64** |
| **Polyuns. fat** | c / py | 226 / 18,331 | 218 / 18,752 | 220 / 18,959 | 194 / 19,165 | 231 / 18,993 |  |
|  | Basic | 1.00 | 0.97 (0.80-1.16) | 1.00 (0.83-1.21) | 0.87 (0.72-1.06) | 1.08 (0.90-1.29) | 0.81 |
|  | **Full** | **1.00** | **0.95 (0.79-1.14)** | **0.97 (0.80-1.17)** | **0.85 (0.70-1.03)** | **1.05 (0.87-1.27)** | **0.98** |
| **n-3 fatty acids** | c / py | 200 / 19,053 | 199 / 18,914 | 232 / 18,809 | 221 / 18,869 | 237 / 18,556 |  |
|  | Basic | 1.00 | 1.01 (0.83-1.22) | 1.11 (0.92-1.35) | 1.05 (0.86-1.27) | 1.05 (0.87-1.27) | 0.52 |
|  | **Full** | **1.00** | **1.00 (0.82-1.21)** | **1.10 (0.91-1.33)** | **0.97 (0.80-1.18)** | **1.02 (0.84-1.24)** | **0.92** |
| **Long-chain n-3** | c / py | 175 / 19,250 | 230 / 18,647 | 240 / 18,730 | 222 / 18,856 | 222 / 18,717 |  |
|  | Basic | 1.00 | 1.22 (1.00-1.49) | 1.24 (1.02-1.50) | 1.10 (0.90-1.34) | 1.03 (0.84-1.26) | 0.66 |
|  | **Full** | **1.00** | **1.24 (1.02-1.51)** | **1.22 (1.00-1.48)** | **1.09 (0.89-1.33)** | **1.03 (0.84-1.26)** | **0.59** |
| **n-6 fatty acids** | c / py | 228 / 18,274 | 223 / 18,514 | 208 / 19,031 | 201 / 19,230 | 229 / 19,151 |  |
|  | Basic | 1.00 | 1.02 (0.85-1.23) | 0.95 (0.79-1.15) | 0.93 (0.77-1.13) | 1.10 (0.91-1.32) | 0.65 |
|  | **Full** | **1.00** | **1.02 (0.85-1.23)** | **0.95 (0.78-1.15)** | **0.94 (0.78-1.14)** | **1.10 (0.91-1.32)** | **0.62** |
| **Protein** | c / py | 243 / 18,516 | 222 / 18,823 | 234 / 18,773 | 195 / 19,072 | 195 / 19,017 |  |
|  | Basic | 1.00 | 0.91 (0.76-1.09) | 1.00 (0.83-1.20) | 0.84 (0.70-1.02) | 0.90 (0.74-1.09) | 0.19 |
|  | **Full** | **1.00** | **0.92 (0.77-1.11)** | **1.07 (0.89-1.28)** | **0.88 (0.72-1.07)** | **0.90 (0.73-1.10)** | **0.28** |

1. Cases / person years. [↑](#footnote-ref-2)
2. Basic model: Adjusted for age, method version, total energy intake (continuous), and season. [↑](#footnote-ref-3)
3. Full model: Adjusted for age, method version, total energy intake (continuous), season, BMI class, smoking category, education, alcohol category, systolic blood pressure, antihypertensive treatment, antihyperlipidemic treatment, leisure time physical activity (quartiles) and quintiles of energy-adjusted dietary fiber. There are only 8,038 men and 1077 cases in the full model due to missing values. [↑](#footnote-ref-4)
